# Comparison of Immune Cell Transfection by Different Vaccine Vectors After Intradermal Injection

**DOI:** 10.3390/vaccines14020185

**Published:** 2026-02-16

**Authors:** Jiani Liu, Destin T. Hinson, Michael J. Hansen, Virginia P. Van Keulen, Brian J. Parrett, Larry R. Pease, Michael A. Barry

**Affiliations:** 1 Department of Medicine, Division of Public Health, Infectious Diseases, and Occupational Medicine, Mayo Clinic, Rochester, MN 55905, USA; 2 Department of Immunology, Mayo Clinic, Rochester, MN 55905, USA; 3 Department of Molecular Medicine, Mayo Clinic, Rochester, MN 55905, USA

**Keywords:** genetic vaccine, lipid nanoparticles, mRNA, DNA, adenovirus, immune cell engineering

## Abstract

**Background/Objectives**: Antigen presenting cells (APCs) and immune cells have unique properties to drive or suppress immune responses. They are therefore key targets for the expression of vaccine antigens or transgene proteins. To better determine the utility of different molecular therapies to modify these cells, mRNA and DNA-based molecular therapy vectors were compared for their ability to genetically modify immune cells after intradermal injections in mice. DNA-based vectors included naked plasmid DNA, plasmid packaged in lipid nanoparticles (LNPs), and replication-defective adenovirus (Ad) vectors. mRNA delivery was mediated by packaging into LNPs like those used in COVID-19 vaccines. **Methods**: Each vector was used to deliver Cre recombinase into Cre reporter mice whose cells were activated to express green fluorescent protein (GFP) and firefly luciferase after Cre recombination. The mice were injected intradermally (ID) near the base of their tail at a site that drains into the inguinal lymph node. Luciferase activity was imaged in the living mice 1 or 4 days after vector injection. The animals were then euthanized, and luciferase activity was imaged in the draining inguinal lymph node. Cells were prepared from the intradermal injection site and from the draining lymph node to determine which immune cells were genetically modified by phenotyping CD45, CD3, and CD11b GFP-positive cells by flow cytometry. Given that the skin uniquely contains Langerhans dendritic cells, these CD207^+^ cells were also phenotyped in skin samples and in the draining lymph node. **Results**: In both the skin and in the draining lymph node, the rank order of luciferase and GFP activation by the vectors were: (1) Ad; (2) mRNA-LNP; (3) DNA-LNP; and (4) naked DNA. Only mRNA-LNP and Ad vectors mediated obvious luciferase activity in the living animals and in the draining lymph nodes by imaging. Notably, both vectors appeared to leak from the ID injection site and not only modify the draining lymph node but also strongly modify the livers of the mice. Naked DNA and DNA-LNP mediated detectable GFP activation in the skin and draining lymph node in some mice, but this activity was low and did not reach statistical significance when compared to PBS-treated animals. mRNA-LNPs and Ad both mediated significant Cre delivery in CD45^+^, CD3^+^, CD11b^+^, and CD207^+^ immune cells in the skin and in the lymph node, with adenovirus mediating consistently higher levels of expression in all of the tested cells. **Conclusions**: These data indicate that mRNA-LNP and Ad vectors mediate stronger modification of skin and lymph node immune cells after intradermal injections. Naked DNA and DNA-LNPs were markedly less potent at this activity than the other vectors. These data are consistent with the higher vaccine potency of mRNA-LNP and Ad vectors and suggest that approaches that increase targeting of immune cell subsets may have utility to increase efficacy while also reducing off-target modification of tissues like the liver.

## 1. Introduction

The immune system can protect one from pathogens or from cancer but can also drive anti-host immune responses to cause disease. Therefore, modulating the activity of immune cells is key to generating robust vaccine responses and to blunt pathogenic immune responses stimulated by an overactive immune system. One approach to modulate the immune system is to transiently or permanently genetically modify immune cells to activate or inhibit their responses with RNA or DNA therapeutics.

Many lessons were learned with RNA and DNA therapeutics and the immune system during the COVID-19 pandemic [1]. Many different vaccines were deployed and were administered by diverse vaccination routes in efforts to control and contain SARS-CoV-2. mRNA-lipid nanoparticle (LNP) vaccines expressing the SARS-CoV-2 spike protein were advanced by Moderna and Pfizer, and these became the numerically favored vaccine during the pandemic [1]. While these were rolled out quickly and show great merits, other vaccines like DNA-based adenovirus (Ad) vaccines were also deployed globally. Ad vaccines were initially deployed more broadly in developed and less developed countries owing to their lower cost, but they became disfavored due to early observations of vaccine-induced thrombotic thrombocytopenia (VITT) [2,3].

The disparate clinical profiles of these platforms stem from fundamental differences in their design and mechanism of action. mRNA-LNP vaccines deliver translated mRNA directly into the cytosol for transient, high-level antigen production [4]. In contrast, adenoviral vectors deliver DNA to the nucleus, often leading to more persistent transgene expression but also carrying risks associated with genomic integration and robust innate immune activation [5]. Non-viral DNA vectors, while safer, face the dual barrier of cellular and nuclear entry, often resulting in lower transfection efficiency [6].

While VITT is a rare but serious side effect associated with adenovirus vector vac-cines, it is worth noting that mRNA vaccines are not without their own risks. Data from the U.S. Vaccine Adverse Event Reporting System (VAERS) showed that mRNA vaccine-associated cardiomyo-pathy in young males occurred at a reported rate of approximately 105.9 cases per million mRNA doses. A consensus document from the European Society of Cardiology (ESC) [7] confirmed that mRNA vaccine myocarditis risk in young males after the second dose can reach over 100 cases per million mRNA doses. For comparison, the incidence of VITT is estimated to be 3 to 15 cases per million adenovirus vector vaccinations [8]—a range that is substantially lower than the mRNA vaccine-associated myocarditis risk observed in young male recipients. In other words, while much attention has focused on VITT, the frequency of this complication is actually lower than that of mRNA vaccine-associated myocarditis in the most affected demographic. Both vaccine types have rare side effects, but the numbers do not suggest that adenovirus vaccines carry a greater burden of risk.

Potency is also a significant consideration in selecting vaccines. Of the most utilized COVID-19 vaccines, only the Johnson and Johnson Ad26 vaccine was given as a single immunization. All others required at least two immunizations as part of their clinical regimen. This need for one versus two vaccinations in the simplest comparison suggests that this DNA-based adenoviral vaccine might have more intrinsic potency than other vaccines that need two or more immunizations.

There seems to be some merit to this conclusion. For example, in the BOOST study, volunteers received the BNT162b2 or mRNA-1273 mRNA vaccines two times, or they received the Ad26.COV2.S vaccine only once [9]. Neutralizing antibody (NAb) titers at 1 and 6 months showed that nABs declined in recipients of BNT162b2 and mRNA-1273, while nABs in recipients of Ad26.COV2.S showed a significant increase. At 6 months, NAbs were significantly higher in the single vaccination Ad26.COV2.S cohort than in the BNT162b2 or mRNA-1273 groups [9].

While the Ad26 vaccine was given once, the similar ChAdOx1 was given two times like the two mRNA vaccines. When ChAdOx1 was compared to the mRNA vaccines in the ZOE COVID Study, SARS-CoV-2 NAbs waned over 5 months in the mRNA groups but climbed in the Ad group over the same time period [10].

These data demonstrate that mRNA and DNA-based Ad vaccines have both their own strengths and their own particular side effects, so both should be considered in future vaccine strategies.

**Figure 1:**
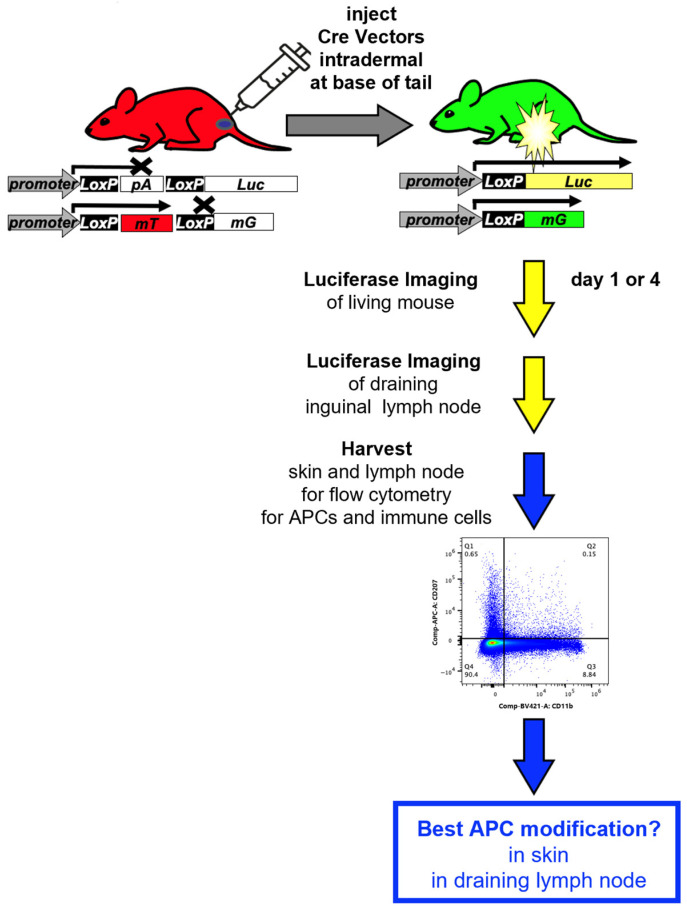
Cartoon of Cre reporter mice and their use in this study to track gene and mRNA delivery to immune cells.

A critical, yet underexplored, metric for directly comparing these platforms is their intrinsic in vivo delivery efficiency—the ability to successfully transfer genetic cargo into the cytoplasm or nucleus of target cells, which is the essential first step preceding antigen expression and immune activation [11]. Given this, the current study aimed to compare and benchmark different vaccine platforms for their ability to deliver transgenes into antigen presenting cells in vivo. To quantify the cell specificity of vector delivery to antigen presenting cells (APCs), we utilized a reporter system; we developed that “fingerprints” gene delivery in vivo by monitoring luciferase (Luc), green fluorescent protein (GFP), and red fluorescent protein (RFP) expression in the same mouse (Figure 1 and [12]). This sensitive genetic switch system allows for the permanent labeling of any cell that has ever been transfected, providing a rigorous measure of delivery ‘breadth’ at single-cell resolution, beyond what transient expression assays can capture [13]. Here, we focused on APC modification, since these are key to the initiation of immune responses. Recognition and presentation of foreign antigens is performed by a variety of antigen-presenting cells (APCs), including macrophages, B cells, and a variety of phenotypically diverse dendritic cells (DCs) [14,15,16,17,18]. Immature DCs can mediate antigen uptake in peripheral organs or in lymph nodes. In the skin are epidermal-resident DCs called Langerhans cells (LCs) [16]. LCs surveil environmental antigens and pathogens that pierce the epidermis of the skin. Once LCs capture and process antigens, these DCs migrate to draining lymph nodes where they mature into DCs that can present antigens to T or B cells [18]. Antigens can also themselves be transported to draining lymph nodes via lymphatics, where they can be sampled by APCs and processed for presentation to T and B cells.

While intradermal vaccines are intentionally injected into the skin to interact with LCs and other APCs, it should be noted that any of the many intramuscular (IM) vaccines also coincidentally deliver antigens into the skin since the needles for IM injection must pierce the skin to reach the muscle. Therefore, LCs and the skin may actually play a role in not only ID but also in IM vaccine strategies. This study focused on the intradermal route; future work comparing the impact of different administration routes (e.g., intramuscular vs. intradermal) on immune cell transfection and systemic spread would be valuable.

To track APC modification efficiently in this study, we compared these vectors by the intradermal (ID) route. This route allowed the surface site of injection to be readily observed. It also allowed the known inguinal draining lymph node for the injection site to also be easily imaged and its cells to be examined.

## 2. Materials and Methods

### 2.1. Vectors

Cre recombinase cDNA was expressed from pTRS-CBh-Cre plasmid DNA [12], or an E1-deleted adenovirus serotype 5 vector [19], or as an mRNA produced by in vitro transcription (IVT) from a T7 Cre recombinase plasmid. Plasmids were purified on Endotoxin-free Maxiprep kits (Machery-Nagel). Adenovirus was produced from 10 Plate CellStacks (Corning) by banding on two CsCl gradients as in [20].

### 2.2. In Vitro Transcription (IVT) of mRNA

The T7-Cre recombinase plasmid was linearized and purified by phenol/chloroform/isoamyl alcohol extraction followed by ethanol precipitation as in [21,22]. This was used as a template for IVT using a T7 RNA synthesis kit (New England Biolabs HiScribe E2040S). The mRNA was synthesized with N1-Methylpseudouridine-5′-Triphosphate (m1ΨTP, Trilink N-1081) and co-transcriptionally capped with Trilink CleanCap reagent AG N-7113. The DNA template was removed with DNase I, and mRNA was purified with NEB Monarch RNA Cleanup Kit T2050. mRNA was assessed by agarose gel electrophoresis and nanodrop.

### 2.3. Lipid Nanoparticle Assembly

Lipid nanoparticles were formulated as in [21,22] using cholesterol (Sigma C3045), 1,2-dimyristoyl-rac-glycero-3-methoxypolyethylene glycol-2000 (DMG-PEG2000, Avanti polar lipids 880151P), 1,2-distearoyl-sn-glycero-3-phosphocholine (DSPC, Avanti polar lipids 850365P), and dilinoleylmethyl-4-dimethylaminobutyrate (D-Lin-MC3-DMA or “MC3”, MedChemExpress HY-112251) on a NanoAssemblr Ignite (Cytiva) microfluidics system. Concentrated LNPs were filtered with a 0.2 µM syringe filter (Pall Acrodisc 4602), and size, polydispersity index, and particle concentration were measured using a Zetasizer Advance Ultra Red (Malvern Panalytical ZSU3305). Encapsulation efficiency and total encapsulated mRNA were quantified using a Quant-iT RiboGreen RNA assay kit (Invitrogen R11490). The LNPs characterization size, zeta potential, and encapsulation efficiency are shown in Supplementary Figure S1.

### 2.4. In Vivo Experiments

All the animal experiments were performed at Mayo Clinic and were approved by the Mayo Clinic Animal Care and Use Committee. All the experiments followed the guidance of the Public Health Service Animal Welfare Policy, Animal Welfare Act and the NIH guidelines in the Guide for the Care and Use of Laboratory Animals.

### 2.5. mT/mG: LSL-Lux Cre Reporter Mice

mT/mG mice (aka. B6.129(Cg)-Gt(ROSA)26Sortm4(ACTB-tdTomato,-EGFP)Luo/J strain # 007676) and LSL-Luc mice (aka FVB.129S6(B6)-Gt(ROSA)26Sortm1(Luc)Kael/J mice strain # 005125) were purchased from The Jackson Laboratory (Bar Harbor, ME, USA). mT/mG, and the LSL-Luc mice were crossed as F1 progeny for the experiments involving luciferase imaging and flow cytometry.

### 2.6. Vector Injections and Tissue Harvest

The Cre reporter mice were injected intradermally on the right side at the base of the tail with the indicated vectors (1) RD-Ad5-Cre (5 × 10^10^ vp); (2) Cre mRNA-LNPs (159 µg/mL); (3) Cre plasmid DNA-LNPs (93.5 µg/mL); and (4) naked Cre plasmid DNA (93.5 µg/mL) with total volume 50 µL. The solvent PBS was injected as a control group in the same volume 50 µL. The Cre plasmid DNA-LNPs and naked Cre plasmid DNA used the same pTRS-CBh-Cre plasmid. The Cre plasmid DNA-LNPs and naked Cre plasmid DNA used the same pTRS-CBh-Cre plasmid. The injections were performed using 31G insulin syringes, with successful intradermal delivery confirmed by the formation of a stable, non-bleeding bleb.

Luciferase expression was assessed via bioluminescence imaging at day 4 post-injection. For in vivo whole-body imaging, the mice were anesthetized with isoflurane. D-luciferin potassium salt (150 mg/kg body weight in PBS; GoldBio) was administered via intraperitoneal injection in the lower left abdominal quadrant. After a 15-min incubation for systemic distribution and substrate conversion, a dorsal image was acquired using a Lumina S5 IVIS Spectrum system (Revvity) with the standardized parameters and an exposure time of 15 seconds (determined from a preliminary time-course as optimal for signal linearity without saturation). Immediately after in vivo imaging, the mice were euthanized. For in vivo imaging of the draining lymph node, the mice were placed in the imaging chamber and imaged using the same instrument parameters as for the live animal. Image analysis was performed using Living Image software (v.4.7.3, Revvity). For the in vivo images, a consistent oval region of interests (ROI) was drawn tightly around each isolated organ (skin or lymph node). The color scale within each ROI was quantified. After IVIS imaging, the skin at the injection site and the draining lymph nodes were removed and collected.

Skin Processing: The collected skin tissues were washed with PBS and subsequently digested using the Epidermis Dissociation Kit (mouse; Miltenyi Biotec) to achieve enzymatic separation of the epidermis and dermis. Briefly, subcutaneous fat was carefully scraped off. The tissue pieces were then placed, dermal side down, onto the prepared Enzyme G solution mixture and incubated at 4 °C for 16 h. After incubation, the epidermis was peeled away from the dermis, transferred into Buffer S, and minced into smaller fragments. These fragments were then transferred to the Enzyme P/A mixture and incubated in a 37 °C water bath for 20 min. The enzymatic reaction was halted by the addition of PB buffer (PBS containing 0.5% bovine serum albumin). The epidermal pieces were mechanically dissociated using a gentleMACS Dissociator (Miltenyi). The resulting cell suspension was passed through a 70 µm MACS SmartStrainer and washed with PB buffer. After discarding the strainer, the filtrate was centrifuged at 300× *g* for 10 min at room temperature. The cell pellet was resuspended in PB buffer for subsequent flow cytometry analysis.

Lymph Node Processing: The right inguinal lymph node was placed onto a 100 µm cell strainer and gently disaggregated using the plunger end of a syringe. The strainer was rinsed with PBS to recover cells. The cell suspension was then passed through a 40 µm cell strainer and rinsed again with PBS. Cells were collected by centrifugation at 800× *g* for 5 min at 4 °C. The supernatant was discarded, and the cell pellet was washed twice with PBS before final resuspension in 2 mL of PBS for further analysis.

### 2.7. Flow Cytometry

Single-cell suspensions were prepared from mouse skin and inguinal lymph nodes. The cells were washed and resuspended in staining buffer (PBS supplemented with 2% FBS). Cell viability was assessed using Ghost Violet 510 Viability Dye (Tonbo Biosciences, San Diego, CA, USA) according to the manufacturer’s instructions. After viability staining, the cells were incubated with fluorochrome-conjugated monoclonal antibodies for surface marker staining for 30 min at 4 °C in the dark. The following antibodies, anti-mouse CD11b (BV421) and CD207/Langerin (APC), were from BioLegend (San Diego, CA, USA). Anti-mouse CD45 (PerCP-Cy5.5) and CD3 (PE-Cy7) were from Tonbo Biosciences. After staining, the cells were fixed with 4% paraformaldehyde (PFA) and incubated on ice overnight, then washed twice with staining buffer and resuspended for acquisition.

Gating Strategy and Specificity Controls: To ensure the specificity of GFP+ signal detection, a stringent gating strategy was employed. The GFP-positive gate was established based on the fluorescence profile of cells from uninjected control mice and PBS-injected control mice, which defined the background autofluorescence and non-specific staining. This same gate was then applied consistently to all experimental samples. To minimize spectral spillover, fluorescence compensation was meticulously performed using UltraComp eBeads™ (Invitrogen) and single-stained cell samples for each fluorochrome in the panel. While a dedicated channel for the constitutive membrane-targeted tdTomato (RFP) reporter was not included in this panel, the near-complete absence of GFP+ events in the negative controls, coupled with the clear separation of signals in experimental groups, provides high confidence that the reported GFP+ populations specifically indicate Cre-mediated recombination and are not artifacts of fluorescence spillover or background. Fluorescence minus one (FMO) controls were included for the key marker CD207.

Data were acquired on a Cytek northern lights spectral flow cytometer (Cytek Biosciences, Fremont, CA, USA). Spectral unmixing was performed using single-stained reference controls, prepared with either antibody captured beads or cells. Flow cytometry data were analyzed using FlowJo software. Doublets were excluded by FSC-A versus FSC-H gating, and dead cells were excluded based on Ghost Violet 510 staining. Immune cells were identified as CD45^+^, followed by analysis of CD3^+^ T cells, CD11b^+^ cells, and CD207^+^ dendritic cell and GFP^+^ subsets. The experiments were performed three times independently (lymph nodes, *n* = 6; for skin tissues, *n* = 4).

### 2.8. Statistical Analysis

The flow cytometry data are presented as percentages of immune cell subsets. Statistical analysis for this study was performed using GraphPad Prism version 10.4.1.3. For comparisons among multiple groups, the nonparametric Kruskal–Wallis test followed by Dunn’s multiple comparisons test was applied, with all pairwise group comparisons performed. The data are presented as individual points representing each mouse, with median and interquartile range (IQR). A *p*-value < 0.05 was considered statistically significant.

## 3. Results

### 3.1. Cre Reporter Mice

Cre reporter mice were used to track and “fingerprint” gene and mRNA delivery in vivo (Figure 1 and [12]). These mice are transgenic in the Rosa26 locus for Cre-activated reporter genes. In LSL-Luc mice, firefly luciferase’s expression is blocked by a LoxP-flanked (floxed) poly-adenylation (PolyA) cassette between the CAG promoter and the luciferase gene (Figure 1).

**Figure 2:**
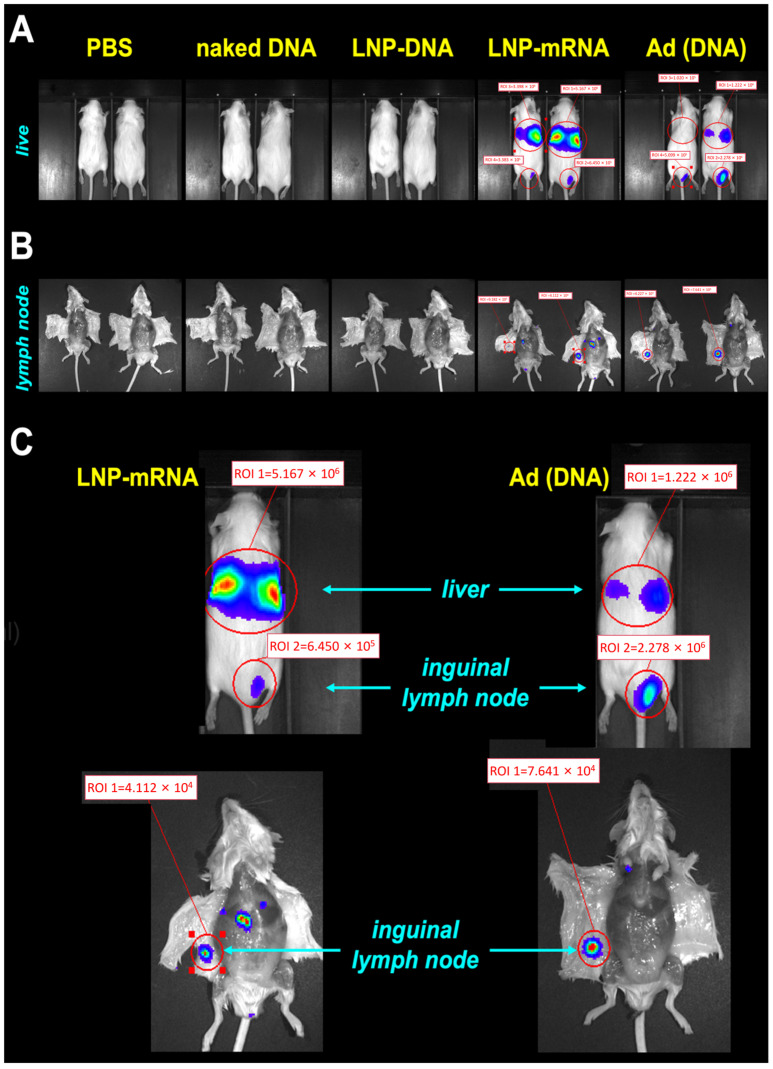
Luciferase imaging of Cre gene delivery after intradermal injections in Cre reporter mice. (**A**) Live animal imaging 4 days after injection with the indicated vectors. (**B**) Imaging of the inguinal lymph nodes in the animals after sacrifice. (**C**) Larger views of in vivo imaging of the injection site skin (upper panels, prone position) and the inguinal lymph nodes (lower panels, supine position) from representative mice injected with mRNA-LNP or Ad vectors from panel A. These experiments were repeated three times, and the images are representative of all the groups.

In the absence of Cre expression, no luciferase is expressed. When Cre is delivered, it deletes the floxed PolyA to activate luciferase (Figure 2 and [12]). mT/mG mice instead have a floxed membrane-targeted red fluorescent protein mTomato (mT) cDNA followed by membrane-targeted GFP (mG) cDNA also in the Rosa26 locus (Figure 1).

In the absence of Cre, mT is expressed on the membranes of all cells in the mouse. When Cre is delivered, mT is deleted and membrane-targeted GFP is expressed [12]. These membrane-targeted reporter proteins provide precise discrimination of which cells are modified by vectors when examined by confocal microscopy. When LSL mice are crossed with mT/mG mice, these hybrid mice have exactly one copy of Cre-activatable luciferase and exactly one copy of Cre-activatable mT/mG. This allows (1) in vivo imaging, (2) cell-specific transduction monitoring via mG, and (3) on/off confirmation of transduction by coordinated loss of mT. The high sensitivity of Cre-loxP-based reporter systems for tracking nucleic acid delivery in vivo is well established. For example, quantitative comparison has demonstrated that detection via Cre-mediated activation of a fluorescent reporter is approximately 8-fold more sensitive than monitoring the direct expression of a co-delivered fluorescent protein [23]. This established sensitivity underscores the utility of such reporter systems, including the one employed here, for the precise mapping of gene or mRNA delivery to target cells.

### 3.2. In Vivo Luciferase Imaging

Cre reporter mice were injected intradermally (ID) with RNA and DNA-based Cre expression vectors at the base of their tail at a site that is drained by the inguinal lymph node. Four days later, the living animals were imaged for luciferase activity on an IVIS (Figure 2A). Naked plasmid DNA and DNA in LNPs did not produce detectable luciferase activity under these conditions, In contrast, mRNA in LNPs and DNA-based Ad Cre vectors both produced IVIS signals (Figure 2A,C). Both vectors produced luciferase at the ID site of injection. Notably, both vectors also produced stronger luciferase activity in the livers of the mice, consistent with prior observations that both vectors leak into the blood [21,24]. The animals were euthanized, and their inguinal lymph nodes were visualized on the underside of their abdominal skin (Figure 2B,C). Imaging demonstrated visible luciferase activity in the nodes by the mRNA-LNP and Ad vectors but not by the others.

### 3.3. Cre Delivery to Immune Cells in the Skin and in the Draining Lymph Node

**Figure 3:**
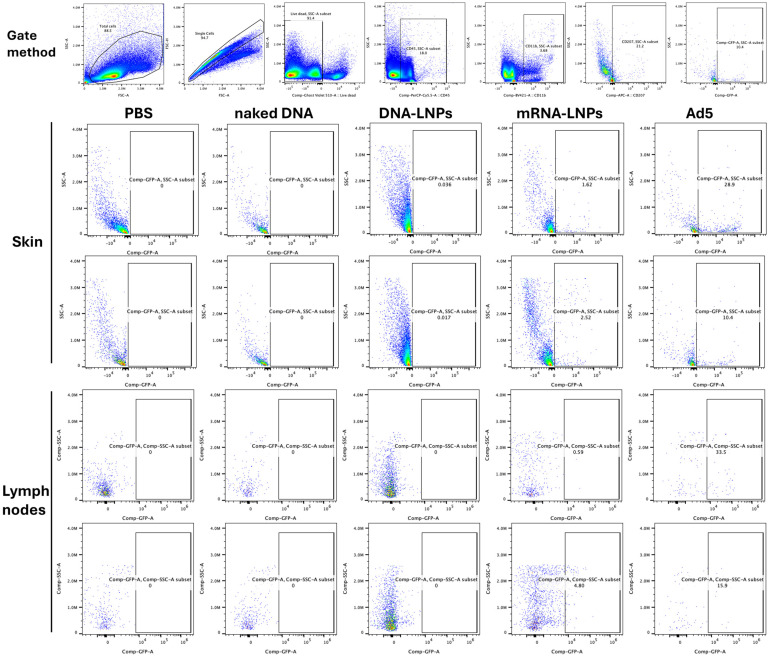
Flow cytometry for Cre-expressing Langerhans dendritic cells in the skin and lymph nodes. The ID site and draining lymph node were harvested 4 days after injection with the indicated vectors, and their cells were analyzed by flow cytometry. Shown are representative scatter plots of GFP-expressing CD45^+^, CD11b^+^, CD207^+^ Langerhans cells. Gating strategy: FSC/SSC → singlets → live (Ghost Violet 510^−^) → CD45^+^ (PerCP-Cy5.5) leukocytes→ CD11b^+^ (BV421)→ CD207^+^ (APC) dendritic cells, followed by subdivision based on GFP^+^ expression. Other immune cell populations were identified based on their lineage-specific surface markers using similar gating.

Skin injection sites and the inguinal lymph nodes were harvested at day 4, and immune cells were isolated from each. These cells were stained with cell phenotyping antibodies against CD45 to identify immune cells, CD3 to identify T cells, CD11b to observe antigen-presenting cells, and with CD207 to identify skin Langerhans DCs in the skin and that had migrated to the draining lymph node. When skin cells were examined, flow cytometry revealed marked activation of mG GFP in Langerhans cells by mRNA-LNP and DNA-based Ad vectors (Figure 3). No Langerhans cells were marked by ID injection of naked DNA. When this same DNA was delivered in LNPs, there was detectable mG activation in Langerhans cells, but this was approximately 100-fold lower than Cre mRNA in LNPs and 300-fold lower than by DNA-based Ad vector.

**Figure 4:**
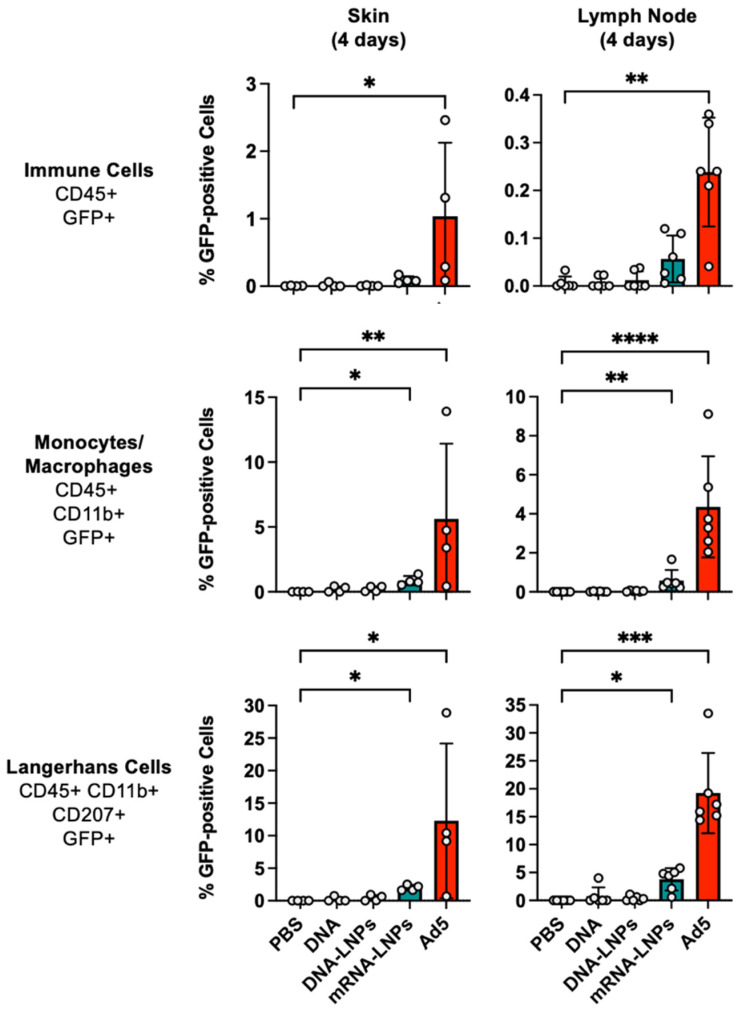
Flow cytometry for Cre-expressing cells in the skin and draining lymph node 4 days after vector injection. Cells were harvested 4 days after injection of vectors, and their cells were analyzed by flow cytometry. Percentages of different immune cell GFP^+^ subsets cell type were analyzed. * *p* < 0.05, ** *p* < 0.01, *** *p* < 0.001, **** *p* < 0.0001 by Kruskal–Wallis test with Dunn’s multiple comparisons.

When Langerhans cells and other immune cells were examined in the skin and draining lymph node, it was apparent that the mRNA-LNP and Ad vectors were superior to the other injections (Figure 4). In the skin, Ad activated GFP in approximately 1% of all CD45+ immune cells (*p* < 0.05 by Kruskal–Wallis test). CD45^+^GFP^+^ cells were observed in mRNA-LNP treated skin, but this did not reach significance. In the draining lymph node, Ad activated GFP in approximately 0.2% of all CD45^+^ immune cells (*p* < 0.01 by Kruskal–Wallis test). Up to 0.1% of CD45^+^ in the lymph node became GFP^+^ after mRNA-LNP treatment, but this did not reach statistical significance. Occasional CD45^+^GFP^+^ cells were generated by DNA groups, but these were lower than in the mRNA and Ad groups.

Ad activated GFP in 4 to 5% of CD45^+^CD11b^+^GFP^+^ monocyte/macrophages at the injection site and in the draining lymph node (*p* < 0.01 and *p* < 0.0001). mRNA-LNPs activated GFP in approximately 1% of these monocyte/macrophage cells (*p* < 0.05 and *p* < 0.01). GFP^+^ monocyte/macrophages were infrequently observed in the skin.

When Langerhans cells were examined 4 days after injection, mRNA-LNPs activated GFP in 2-5% of these cells in the skin and lymph node (*p* < 0.05). In contrast, Ad activated GFP in up to 30% of these cells in both sites (*p* < 0.05 and 0.001). Rare GFP^+^ Langerhans cells were observed in either site after DNA delivery.

## 4. Discussion

The goal of this work was to map the initial transgene delivery paths of vectors relevant to gene or mRNA-based vaccines. This was tested after intradermal injection to enable simple tracking to the known inguinal draining lymph node. It is important to note that the doses used here—5 × 10^10^ viral particles for Ad5, 7.95 µg for mRNA-LNP, 4.675 µg for DNA-LNP, and 4.675 µg for naked DNA—represent immunologically effective amounts for each platform based on established literature [25,26,27,28,29], rather than equimolar quantities. This approach, common in comparative vector studies [30,31], evaluates performance at respective ‘working doses’ to model real-world vaccine scenarios. Consequently, our conclusions pertain to the relative delivery efficiency under these practical conditions, while acknowledging that direct molar comparisons would require a different experimental design. Under these conditions, naked DNA and DNA in LNPs mediate weak to no gene delivery to cells as determined by luciferase imaging and by flow cytometry. The markedly higher efficacy of mRNA-LNP compared to DNA-LNP likely stems from fundamental biological differences: mRNA requires only cytosolic delivery for translation, whereas DNA must additionally traverse the nuclear membrane for transcription [4,32]. Furthermore, differences in the formulation and colloidal stability of LNPs optimized for encapsulating mRNA versus DNA may influence their cellular uptake and endosomal escape kinetics [33].

In contrast, mRNA delivered LNPs and DNA-based Ad vectors mediated significant delivery in antigen presenting cells and Langerhans cells in the skin. In general, the Ad vector mediated higher genetic modification of immune cells than the mRNA-LNP vector in the skin and in the draining lymph node. This ranking aligns with the known potent immunogenicity of these platforms in clinical settings. It is important to interpret this result within the context of our experimental readout. The Cre-loxP reporter system provides a sensitive, permanent record of any cell that has ever received and expressed the Cre transgene, offering an unparalleled measure of the ‘breadth’ of delivery (i.e., the frequency of successfully transfected cells) [13]. It does not, however, quantify the ‘depth’ of expression (i.e., the amount or duration of protein produced per cell), which is an inherent property of the platform (e.g., transient high expression from mRNA versus sustained expression from Ad vectors) [34]. Our in vivo bioluminescence imaging [35] (Figure 2) provides complementary, whole-organ-level information on total expression intensity. Thus, our study is uniquely positioned to compare the intrinsic capability of each platform to deliver genetic cargo to immune cells—the critical first step in vaccine efficacy. We focused on standard needle injection, the most clinically translatable route, acknowledging that methods like electroporation can enhance DNA vaccine delivery but introduce additional complexity [36,37]. Our findings therefore map the initial delivery landscape upon which subsequent expression and immunogenicity are built.

Notably, both of these robust vectors also mediated off-target delivery to the liver, as evidenced by luciferase imaging in the living animals. A key mechanistic question arising from this finding is the route of systemic dissemination: whether vectors directly entered the bloodstream at the injection site (vascular entry) or were first drained via lymphatics to the node before entering circulation (lymphatic-to-vascular route). The size of our LNPs (~100 nm) is pertinent here, as particles of this dimension are capable of both direct vascular entry and efficient lymphatic drainage. Regardless of the initial pathway, systemically circulating nanoparticles in this size range are known to accumulate in the liver due to filtration by hepatic sinusoids and uptake by resident macrophages. Our experimental design, with a primary endpoint at Day 4, captures the net outcome of systemic spread but cannot delineate the precise anatomical pathway. Future studies employing early time-point pharmacokinetics or lymphatic blockade models are required to resolve this [38,39]. Regardless of the pathway, our data unequivocally demonstrate that even localized intradermal administration can lead to significant systemic exposure and transfection of distal organs like the liver. Our flow cytometry gating strategy, defined using stringent uninjected and PBS-injected controls, confirmed that the reported GFP+ signals were specific to Cre recombination. However, a technical limitation of this study is the absence of cellular phenotyping data from the liver. Furthermore, the permanent nature of the Cre-loxP reporter means that a GFP+ signal in the liver could result from either the direct transfection of hepatic resident cells (e.g., hepatocytes or Kupffer cells) or the migration of immune cells that were originally transfected in the skin or lymph node. Our current experimental design cannot distinguish between these two fundamentally different cellular origins of the signal. The strong bioluminescent signal indicates transfection events within the organ, but the precise identity of the transfected cells (e.g., hepatocytes versus Kupffer cells) and the contribution of direct transfection versus migration of peripherally transfected immune cells remain to be determined in future studies. This off-target delivery was not unexpected for either vector and might explain to some degree certain side effects during genetic immunization. For adenoviral vectors, systemic distribution has been linked, in rare instances, to serious adverse events such as vaccine-induced immune thrombotic thrombocytopenia (VITT) [40,41], underscoring the importance of understanding the in vivo biodistribution and developing safer, more targeted next-generation vectors. Similarly, the hepatic tropism of some LNP formulations is a known factor that can influence both immunogenicity and reactogenicity [42,43], a phenomenon likely influenced by their physicochemical properties such as the particle size observed in our study (~100 nm, Supplementary Figure S1). Our findings directly visualize this systemic leakage, reinforcing that even localized intradermal administration does not guarantee strictly local effects.

This study has certain limitations that point to valuable future directions. First, while our comparison reveals clear differences in delivery efficiency at standardized, immunologically effective doses, the inherent structural disparities between platforms preclude a direct molar equivalence comparison—a common challenge in the field [31]. Second, our experimental timeline was designed to capture the peak of initial antigen delivery and immune cell trafficking (Days 1–4), a critical period for innate immune activation and antigen presentation [44,45]. Consequently, systematic data on long-term transgene persistence and vector clearance, particularly relevant for interpreting the off-target liver signal we observed, were not obtained. Finally, the identity of the transfected cells within the liver remains to be determined at a single-cell level. Resolving these questions—through extended time-course studies and detailed cellular phenotyping—will be essential to fully understand the translational safety and durability profiles of these promising vaccine platforms [46].

## 5. Conclusions

In summary, our direct in vivo comparison highlights a clear hierarchy in the intrinsic ability of vaccine platforms to deliver genes to skin and lymph node immune cells via a standard intradermal route. The stark contrast between mRNA/Ad platforms and DNA platforms under these conditions provides a cellular delivery rationale for their differing clinical immunogenicity. Simultaneously, the non-negligible off-target delivery to sites like the liver, visualized here, provides a mechanistic insight into potential systemic effects. Our study thus provides a sensitive in vivo map of transfection events (“event detection”), establishing a critical foundation. For the observed off-target delivery, the next frontier lies in achieving similar cellular resolution within organs like the liver—to pinpoint the exact cell types transfected and their mechanistic origins (direct transfection vs. cell migration)—which will be pivotal for tailoring next-generation vectors with optimal tissue specificity and safety profiles.

## Data Availability

Data will be provided upon request.

## Supplementary Materials

The following supporting information can be downloaded at: https://www.mdpi.com/article/10.3390/vaccines14020185/s1, Figure S1: Characterization of Lipid Nanoparticles (LNPs); Figure S2: Luciferase Imaging of Cre Gene Delivery after Intradermal Injections in all the Cre Reporter Mice; Figure S3: Luciferase Imaging and Flow Cytometry of Cre Gene Delivery after Intradermal Injections in all the Cre Reporter Mice.

## Abbreviations

The following abbreviations are used in this manuscript:

Ad	adenovirus
APC	antigen presenting cell
ID	intradermal
GFP	green fluorescent protein
Luc	luciferase
mG	membrane targeted GFP
RFP	red fluorescent protein
mT	membrane targeted mTomato RFP
VAERS	vaccine adverse event reporting system
VITT	vaccine-induced thrombotic thrombocytopenia

## References

[1] Prubeta B.M. (2021). Current State of the First COVID-19 Vaccines. Vaccines.

[2] Greinacher A., Langer F., Makris M., Pai M., Pavord S., Tran H., Warkentin T.E. (2022). Vaccine-induced immune thrombotic thrombocytopenia (VITT): Update on diagnosis and management considering different resources. J. Thromb. Haemost..

[3] Rodriguez E.V.C., Bouazza F.Z., Dauby N., Mullier F., d’Otreppe S., Jissendi Tchofo P., Bartiaux M., Sirjacques C., Roman A., Hermans C. (2022). Fatal vaccine-induced immune thrombotic thrombocytopenia (VITT) post Ad26.COV2.S: First documented case outside US. Infection.

[4] Sahin U., Karikó K., Türeci Ö. (2014). mRNA-based therapeutics—Developing a new class of drugs. Nat. Rev. Drug Discov..

[5] Lee C.S., Bishop E.S., Zhang R., Yu X., Farina E.M., Yan S., Zhao C., Zheng Z., Shu Y., Wu X. (2017). Adenovirus-Mediated Gene Delivery: Potential Applications for Gene and Cell-Based Therapies in the New Era of Personalized Medicine. Genes. Dis..

[6] Yin H., Kanasty R.L., Eltoukhy A.A., Vegas A.J., Dorkin J.R., Anderson D.G. (2014). Non-viral vectors for gene-based therapy. Nat. Rev. Genet..

[7] Heidecker B., Dagan N., Balicer R., Eriksson U., Rosano G., Coats A., Tschope C., Kelle S., Poland G.A., Frustaci A. (2022). Myocarditis following COVID-19 vaccine: Incidence, presentation, diagnosis, pathophysiology, therapy, and outcomes put into perspective. A clinical consensus document supported by the Heart Failure Association of the European Society of Cardiology (ESC) and the ESC Working Group on Myocardial and Pericardial Diseases. Eur. J. Heart Fail..

[8] Petito E., Gresele P. (2025). VITT Pathophysiology: An Update. Vaccines.

[9] Prather A.A., Dutcher E.G., Robinson J., Lin J., Blackburn E., Hecht F.M., Mason A.E., Fromer E., Merino B., Frazier R. (2023). Predictors of long-term neutralizing antibody titers following COVID-19 vaccination by three vaccine types: The BOOST study. Sci. Rep..

[10] Menni C., May A., Polidori L., Louca P., Wolf J., Capdevila J., Hu C., Ourselin S., Steves C.J., Valdes A.M. (2022). COVID-19 vaccine waning and effectiveness and side-effects of boosters: A prospective community study from the ZOE COVID Study. Lancet Infect. Dis..

[11] Guevara M.L., Persano F., Persano S. (2020). Advances in Lipid Nanoparticles for mRNA-Based Cancer Immunotherapy. Front. Chem..

[12] Hillestad M.L., Guenzel A.J., Nath K.A., Barry M.A. (2012). A vector-host system to fingerprint virus tropism. Hum. Gene Ther..

[13] Muzumdar M.D., Tasic B., Miyamichi K., Li L., Luo L. (2007). A global double-fluorescent Cre reporter mouse. Genesis.

[14] Akyol R., Dalod M. (2025). Identity, Functions, and the Spatiotemporal Maturation of Type 1 Conventional Dendritic Cells. Immunol. Rev..

[15] Vine E.E., Austin P.J., O’Neil T.R., Nasr N., Bertram K.M., Cunningham A.L., Harman A.N. (2024). Epithelial dendritic cells vs. Langerhans cells: Implications for mucosal vaccines. Cell Rep..

[16] Banchereau J., Briere F., Caux C., Davoust J., Lebecque S., Liu Y.J., Pulendran B., Palucka K. (2000). Immunobiology of dendritic cells. Annu. Rev. Immunol..

[17] Melero I., Vile R.G., Colombo M.P. (2000). Feeding dendritic cells with tumor antigens: Self-service buffet or a la carte?. Gene Ther..

[18] Timares L., Takashima A., Johnston S.A. (1998). Quantitative analysis of the immunopotency of genetically transfected dendritic cells. Proc. Natl. Acad. Sci. USA.

[19] Rubin J.D., Nguyen T.V., Allen K.L., Ayasoufi K., Barry M.A. (2019). Comparison of Gene Delivery to the Kidney by Adenovirus, Adeno-Associated Virus, and Lentiviral Vectors After Intravenous and Direct Kidney Injections. Hum. Gene Ther..

[20] Crosby C.M., Barry M.A. (2014). IIIa deleted adenovirus as a single-cycle genome replicating vector. Virology.

[21] Parrett B.J., Yamaoka S., Barry M.A. (2025). Reducing off-target expression of mRNA therapeutics and vaccines in the liver with microRNA binding sites. Mol. Ther. Methods Clin. Dev..

[22] Betageri K.R., Meridew J.A., Parrett B.J., Gilbert R.M., Link P.A., Schussler N.A., Mercado-Perez A., Caporarello N., Barry M.A., Tschumperlin D.J. (2025). Lung-targeted Lipid Nanoparticle Delivery of a Matricellular mRNA Promotes Fibrotic Lung Repair. Am. J. Respir. Cell Mol. Biol..

[23] Deverman B.E., Pravdo P.L., Simpson B.P., Kumar S.R., Chan K.Y., Banerjee A., Wu W.L., Yang B., Huber N., Pasca S.P. (2016). Cre-dependent selection yields AAV variants for widespread gene transfer to the adult brain. Nat. Biotechnol..

[24] Weaver E.A., Nehete P.N., Buchl S.S., Senac J.S., Palmer D., Ng P., Sastry K.J., Barry M.A. (2009). Comparison of replication-competent, first generation, and helper-dependent adenoviral vaccines. PLoS ONE.

[25] Ning W., Qian X., Dunmall L.C., Liu F., Guo Y., Li S., Song D., Liu D., Ma L., Qu Y. (2024). Non-secreting IL12 expressing oncolytic adenovirus Ad-TD-nsIL12 in recurrent high-grade glioma: A phase I trial. Nat. Commun..

[26] Kimball K.J., Preuss M.A., Barnes M.N., Wang M., Siegal G.P., Wan W., Kuo H., Saddekni S., Stockard C.R., Grizzle W.E. (2010). A phase I study of a tropism-modified conditionally replicative adenovirus for recurrent malignant gynecologic diseases. Clin. Cancer Res..

[27] Wu S., Huang J., Zhang Z., Wu J., Zhang J., Hu H., Zhu T., Zhang J., Luo L., Fan P. (2021). Safety, tolerability, and immunogenicity of an aerosolised adenovirus type-5 vector-based COVID-19 vaccine (Ad5-nCoV) in adults: Preliminary report of an open-label and randomised phase 1 clinical trial. Lancet Infect. Dis..

[28] Laczkó D., Hogan M.J., Toulmin S.A., Hicks P., Lederer K., Gaudette B.T., Castaño D., Amanat F., Muramatsu H., Oguin T.H. (2020). A Single Immunization with Nucleoside-Modified mRNA Vaccines Elicits Strong Cellular and Humoral Immune Responses against SARS-CoV-2 in Mice. Immunity.

[29] Gary E.N., Weiner D.B. (2020). DNA vaccines: Prime time is now. Curr. Opin. Immunol..

[30] Lundstrom K. (2018). Viral Vectors in Gene Therapy. Diseases.

[31] Suschak J.J., Williams J.A., Schmaljohn C.S. (2017). Advancements in DNA vaccine vectors, non-mechanical delivery methods, and molecular adjuvants to increase immunogenicity. Hum. Vaccin. Immunother..

[32] Kowalski P.S., Rudra A., Miao L., Anderson D.G. (2019). Delivering the Messenger: Advances in Technologies for Therapeutic mRNA Delivery. Mol. Ther..

[33] Liu M.A. (2019). A Comparison of Plasmid DNA and mRNA as Vaccine Technologies. Vaccines.

[34] Linares-Fernández S., Lacroix C., Exposito J.Y., Verrier B. (2020). Tailoring mRNA Vaccine to Balance Innate/Adaptive Immune Response. Trends Mol. Med..

[35] Sadikot R.T., Blackwell T.S. (2005). Bioluminescence imaging. Proc. Am. Thorac. Soc..

[36] Hirao L.A., Wu L., Satishchandran A., Khan A.S., Draghia-Akli R., Finnefrock A.C., Bett A.J., Betts M.R., Casimiro D.R., Sardesai N.Y. (2010). Comparative analysis of immune responses induced by vaccination with SIV antigens by recombinant Ad5 vector or plasmid DNA in rhesus macaques. Mol. Ther..

[37] Babiuk S., Baca-Estrada M.E., Foldvari M., Storms M., Rabussay D., Widera G., Babiuk L.A. (2002). Electroporation improves the efficacy of DNA vaccines in large animals. Vaccine.

[38] Trevaskis N.L., Kaminskas L.M., Porter C.J. (2015). From sewer to saviour—Targeting the lymphatic system to promote drug exposure and activity. Nat. Rev. Drug Discov..

[39] Oussoren C., Zuidema J., Crommelin D.J., Storm G. (1997). Lymphatic uptake and biodistribution of liposomes after subcutaneous injection. II. Influence of liposomal size, lipid compostion and lipid dose. Biochim. Biophys. Acta.

[40] Greinacher A., Thiele T., Warkentin T.E., Weisser K., Kyrle P.A., Eichinger S. (2021). Thrombotic Thrombocytopenia after ChAdOx1 nCov-19 Vaccination. N. Engl. J. Med..

[41] Baker A.T., Boyd R.J., Sarkar D., Teijeira-Crespo A., Chan C.K., Bates E., Waraich K., Vant J., Wilson E., Truong C.D. (2021). ChAdOx1 interacts with CAR and PF4 with implications for thrombosis with thrombocytopenia syndrome. Sci. Adv..

[42] Hou X., Zaks T., Langer R., Dong Y. (2021). Lipid nanoparticles for mRNA delivery. Nat. Rev. Mater..

[43] Cullis P.R., Hope M.J. (2017). Lipid Nanoparticle Systems for Enabling Gene Therapies. Mol. Ther..

[44] Iwasaki A., Medzhitov R. (2015). Control of adaptive immunity by the innate immune system. Nat. Immunol..

[45] Allan R.S., Waithman J., Bedoui S., Jones C.M., Villadangos J.A., Zhan Y., Lew A.M., Shortman K., Heath W.R., Carbone F.R. (2006). Migratory dendritic cells transfer antigen to a lymph node-resident dendritic cell population for efficient CTL priming. Immunity.

[46] Nayak S., Herzog R.W. (2010). Progress and prospects: Immune responses to viral vectors. Gene Ther..

