# Supplementary material for: Comparison of Immune Cell Transfection by Different Vaccine Vectors After Intradermal Injection
Source: Vaccines (Basel). 2026 Feb 16;14(2):185. doi: 10.3390/vaccines14020185 (PMC12945243; doi:10.3390/vaccines14020185)
Supplement: Supplementary file 1 [file vaccines-14-00185-s001.zip › vaccines-4098437-supplementary.pdf]

Characterization analysis

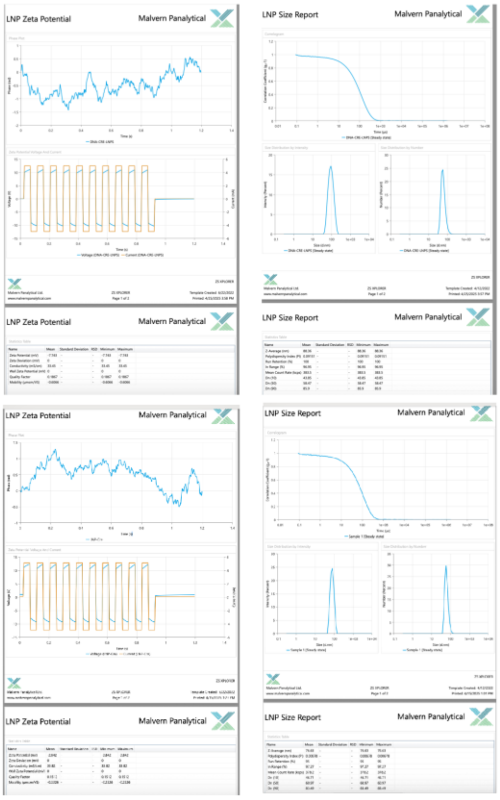

DNA-CRE-LNPs

Sample ID	Encapsulation Efficiency	Total DNA Concentration	Encapsulated DNA Concentration
	%	ug/mL	ug/mL
LNP-Cre DNA	94.3	99.2	93.5

RNA-CRE-LNPs

Sample ID	Encapsulation Efficiency	Total siRNA Concentration	Encapsulated siRNA Concentration
	%	ug/mL	ug/mL
LNP-NLS-Cre	95.0	167.6	159.2

Supplementary Figure S1: Characterization of Lipid Nanoparticles (LNPs), LNP Zeta potiential, Size report, encapsulation efficiency and concentration. Upper: DNA-CRE-LNPs, lower: RNA-CRE-LNPs

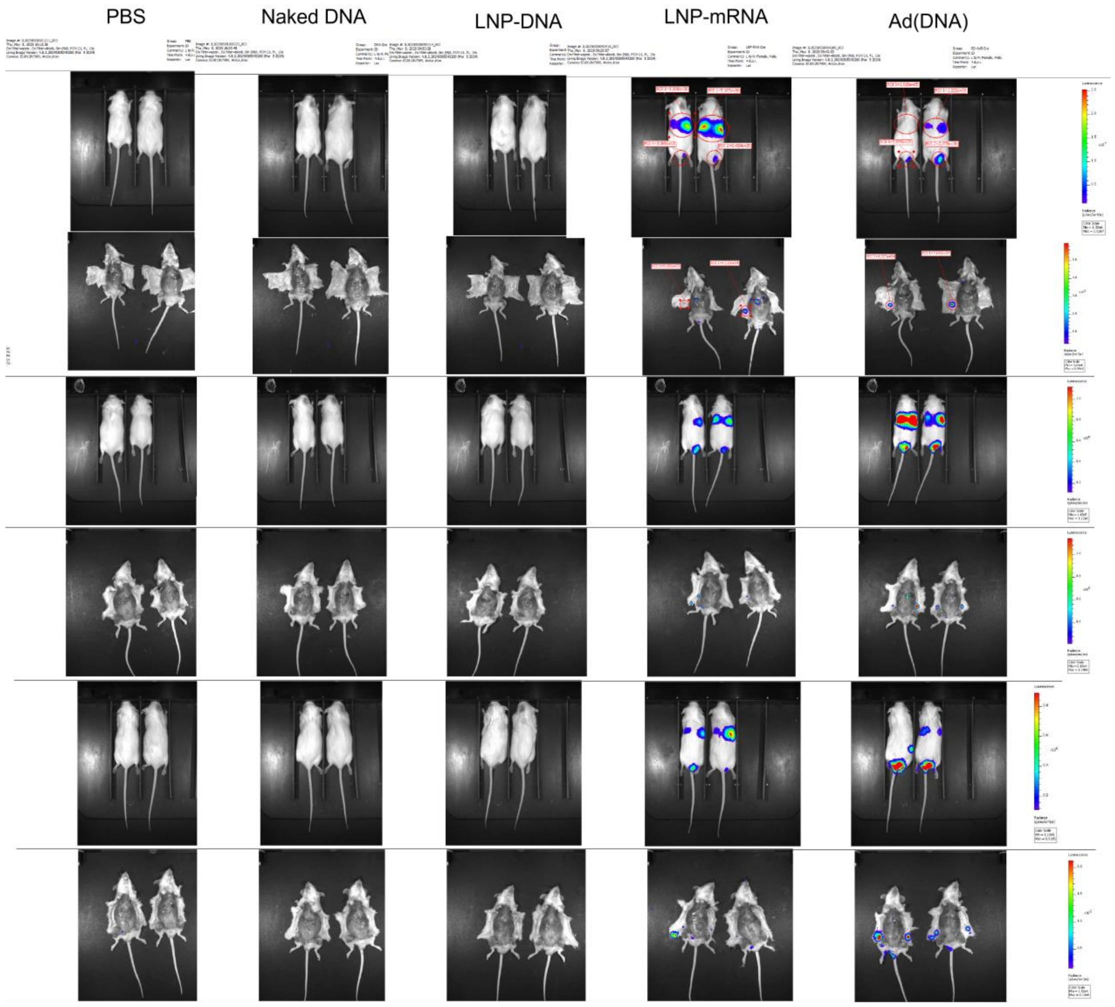

Supplementary Figure S2: Luciferase Imaging of Cre Gene Delivery after Intradermal Injections in all the Cre Reporter Mice. Live animal imaging 4 days after injection with the indicated vectors and imaging of the inguinal lymph nodes in the animals after sacrifice. The injection site skin (upper panels, prone position) and the inguinal lymph nodes (lower panels, supine position). n=6.

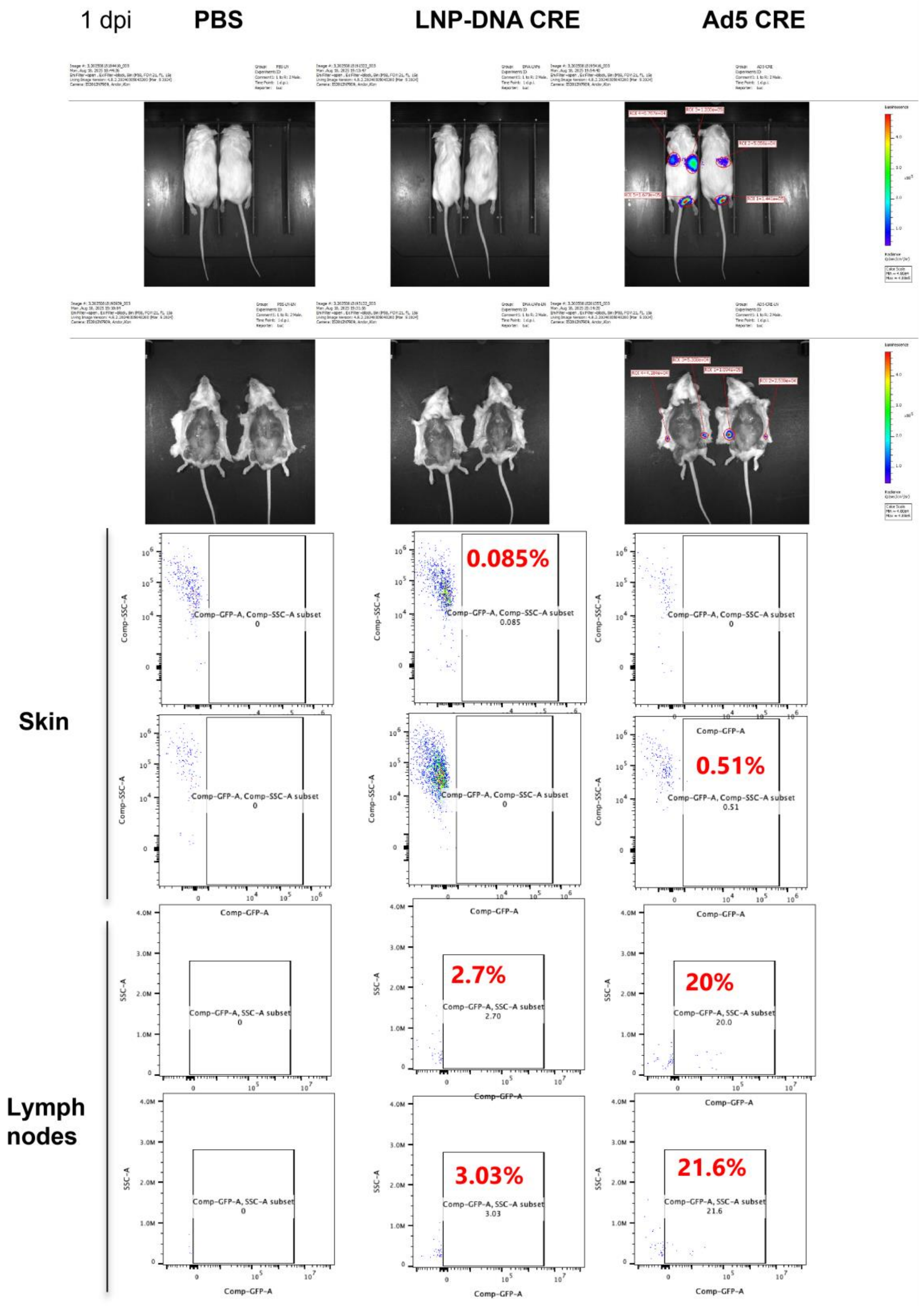

Supplementary Figure S3: Luciferase Imaging and Flow Cytometry of Cre Gene Delivery after Intradermal Injections in all the Cre Reporter Mice. Live animal imaging 1 days after injection with the indicated vectors and imaging of the inguinal lymph nodes in the animals after sacrifice. And the flow Cytometry for Cre-Expressing Langerhans Dendritic Cells in the Skin and lymph nodes. The ID site and draining lymph node were harvested 1 days after

injection with the indicated vectors and their cells were analyzed by flow cytometry.
